# Extracellular Acidification Acts as a Key Modulator of Neutrophil Apoptosis and Functions

**DOI:** 10.1371/journal.pone.0137221

**Published:** 2015-09-04

**Authors:** Shannan Cao, Peng Liu, Haiyan Zhu, Haiyan Gong, Jianfeng Yao, Yawei Sun, Guangfeng Geng, Tong Wang, Sizhou Feng, Mingzhe Han, Jiaxi Zhou, Yuanfu Xu

**Affiliations:** 1 State Key Laboratory of Experimental Hematology, Institute of Hematology and Blood Diseases Hospital, Chinese Academy of Medical Sciences and Peking Union Medical College, Tianjin, China; 2 Hematopoietic Stem Cell Transplantation Center, Institute of Hematology and Blood Diseases Hospital, Peking Union Medical College and Chinese Academy of Medical Sciences, Tianjin, China; University Hospital Basel, SWITZERLAND

## Abstract

In human pathological conditions, the acidification of local environment is a frequent feature, such as tumor and inflammation. As the pH of microenvironment alters, the functions of immune cells are about to change. It makes the extracellular acidification a key modulator of innate immunity. Here we detected the impact of extracellular acidification on neutrophil apoptosis and functions, including cell death, respiratory burst, migration and phagocytosis. As a result, we found that under the acid environment, neutrophil apoptosis delayed, respiratory burst inhibited, polarization augmented, chemotaxis differed, endocytosis enhanced and bacteria killing suppressed. These findings suggested that extracellular acidification acts as a key regulator of neutrophil apoptosis and functions.

## Introduction

Neutrophils are the most abundant cell type among circulating white blood cells and constitute the first line of host defense against invading microorganisms. In response to inflammatory stimuli, neutrophils migrate from the circulation to the tissues, where they protect their host by engulfing, killing, and digesting. However, neutrophils are also double-edged swords of innate immunity. The reactive oxygen species and granule enzymes released by neutrophils can damage the surrounding tissues and cause unwanted and exaggerated inflammation. Hence, the fate of neutrophils must be well controlled.

Since then, the modulation of neutrophil death and functions had been extensively explored. It is reported that the immune response of neutrophils can be modulated by the change of extracellular pH[1, 2]. The acidification of local environment is a frequent feature under some pathological conditions. In an earlier study, the pH of inflammatory exudates was measured, the overall mean pH can decrease to 7.048[3]. Another research aimed to synovial fluid showed a pH falling from 7.0 to 6.0[4]. Wike-Hoole *et al*. summarized several thousand microelectrode measurements of pH in human and animal tumors, there wasa wide range of pH value in malignant tissue, from 5.8 to 7.6[5]. In 1995, Engin K *et al*. carried out a research on 58 patients who had tumors, the result showed an average extracellular pH of 7.06±0.05 (range 5.66–7.78)[6]. Other studiesshowed that the extracellular acidification was related to the process of inflammatory, and the pH value of the microenvironment couldreach to 5.5–7.0[7–9]. Another research demonstrated that severe tissue ischemia induced extracellular acidification[10, 11]. For good or bad, changes in the extracellular pH may induce significant alterations in the immune response.

Studies have indicated that extracellular acidification may change the release of inflammatory mediator, and different kinds of acid at specific pH value can cause a different change[1]. Besides that, extracellular acidification also plays an important role in modulating the function of immune cells, including polymorphonuclear leukocytes, macrophages, and lymphocytes[2, 12]. Acid-treated monocytes and macrophages produce more IL-1beta and trigger NLRP3 inflammasome activation[13, 14]. At the pulmonary airways, acid airway surface liquid attenuates the bacteria killing ability[15, 16]. Beyond the immune system, in the cultured neurons, low pH prevents excitotoxic and ischemic cell death by inhibiting NADPH oxidase[17]. In our study, we focused on the impact of the extracellular acidification on the function of neutrophils, including apoptosis, reactive oxygen species production, ruffling, chemotaxis and phagocytosis, trying to make a systematic understanding on the relationship between the neutrophil functions and their microenvironment.

## Materials and Methods

### Preparation of human neutrophils

Peripheral blood samples were bought from Tianjin Blood Center(China) with EDTA anticoagulation. Neutrophils were purified using a standard protocol. Firstly, erythrocytes were sedimented by adding an equal volume of hetastarch(B. Braun, US)at room temperature for 30 min. The erythrocyte-depleted supernatants were then layered on the lymphocyte separation medium(Ficoll-Hypaque solution, TBDScience) and centrifuged at 2000 rpm at room temperature for 20 min. Contaminated erythrocytes in the neutrophil pellets were lysed after a treatment with red blood lysis buffer(STEM CELL TM Technology, CA). Neutrophils were washed and resuspended in RPMI 1640 medium(Thermo Scientific, US) containing 10% Fetal Bovine Serum(Hyclone) at a density of 3×10^6^ cells per ml and maintained at 37°C.

### Culture medium acidification

RPMI 1640 Medium was added with 10% FBS, and then adjusted separately to pH 6.0, 6.5, 7.0, 7.4 by the addition of 70% HCl. The pH value was detected using pH meter(Eutech, US).

### FACS analysis of neutrophil death

Purified neutrophils were cultured in RPMI 1640 medium with 10% FBS of various pH value. At indicated time point, cells were collected and stained with annexin V-FITC and Propidium Iodide(BD Pharmingen, US). Flow cytometry analysis was performed by BD LSR II Flow Cytometer. We used the freshly purified neutrophil sample as negative control to gate the living cell(S1 Fig). Ten thousand cells were detected and analyzed by BD FACSDiva software.

### Western blot analyse for caspase 3 and p-Akt

Neutrophils were cultured at 3×10^6^ cells per ml in the 1640 medium of different pH. At each indicated time point, 2×10^6^ cells were collected and lysed immediately with 200ul LDS sample buffer(Invitrogen)including protease inhibitor cocktail(Roche). Samples were boiled at 100°C for 5 min and transferred on ice. 25μl of the lysate was used for western blot analysis. In the western blotting, 5%-15% SDS PAGE system was used for protein separation, and ECL western blotting kit (Millipore) was used for protein detection.

### Immunofluorescence staining of cleaved caspase 3

Purified neutrophils were cultured in the medium of pH 6.0 or 7.4 for 24h. Then 5×10^6^ cells were collected and fixed with 4% formaldehyde. Neutrophils were permeabilized with 0.5% Triton-X in PBS for 20 min in room temperature. After preblocking with 5% BSA, cells were stained for 30 minutes with rhodamine phalloidin(Molecular probes). Anti-cleaved caspase 3 antibody (Cell Signaling Technology) and FITC labeled secondary antibodies were used to stain the activated caspase 3. After staining nucleus with DAPI, cells were spread on the slide by centrifuging. Finally we put a coverslip on the slide with antifadent mountant solutions, and observed using a fluorescence microscope(PerkinElmer UltraVIEW Vox).

### Labeling and imaging of ROS

Purified neutrophils(1×10^6^/ml) in PBS were incubated for 15min at 37°C with or without diphenyleneiodonium chloride (DPI, Sigma, US) and then further incubated for 20min with DCFH-DA(Beyotime). After two washes with PBS, neutrophils were resuspended in the prepared acidized or normal medium for 2 hours. 1μM fMLP(Sigma) were used as an agonist. Images were captured using Leica laser scanning confocal microscope. Fluorescence intensity were read by the Microplate Reader(Synergy H4, BioTek)

### ROS measurement

ROS production was measured by a luminol chemiluminescence assay [18]. Neutrophils were resuspended in PBS with 0.1% BSA(3×10^6^/ml) of indicated pH value, 50μM lunimol and 4U/ml horseradish peroxidase(Sigma) were added to the cell suspension. Neutrophils were stimulated with 1μM fMLP, which were added using a computer-programmed injector equipped in the Microplate Reader(Synergy H4, BioTek). The production of ROS is determined by their ability to catalyze the oxidation of luminol, which results in light emission that can be detected by the luminometer.

### Measurement of actin gluthionylation by immunoprecipitation and western blot

Purified neutrophils were cultured in the medium of pH 6.0 or 7.4 for 4h, and then 1×10^7^ cells were collected and lysed in RIPA lysis buffer (Beyotime, 50mM Tris(pH 7.4), 150mM NaCl, 1% NP-40, 0.5% sodium deoxycholate, 0.1% SDS) with protease inhibitor cocktail(Roche). Protein A/G agrose beads(Abmart) were added to the lysate to pre-clearing the IgG protein to reduce non-specific binding. Immunoprecipitation was carried out at 4°C with anti-actin antiboby(Cell Signaling Technology) mixed to the lysate and incubated overnight. And then protein A/G agrose beads were added to pull down the immune complex. After centrifuging, the precipitation was re-suspended with LDS sample buffer(Life Technology), and boiled for 10min at 100°C. Subsequently, we used western blot to detect the glutathionylation of actin with the Anti-Glutathione antibody(Abcam).

### Quantification and Imaging of F-actin levels

Purified neutrophils were cultured in the medium of pH 6.0 or 7.4 for 4h. Then these cells were stimulated with 10nM fMLP for 3min, and fixed with 4% formaldehyde. Neutrophils were permeabilized with 0.5% Triton-X in PBS for 20 min in room temperature. After preblocking with 5% BSA, cells were stained for 30 min with rhodamine phalloidin(Molecular probes). Intensity of phalloidin staining was analyzed using a Microplate Reader(Synergy H4, BioTek). For the imaging of F-actin, neutrophils were seeded on the fibronectin (0.1mg/ml, Sigma) coated coverslips in the acidized or normal medium, and then stimulated, fixed, permeabilized, stained using the same protocol. Finally we put the coverslip with cells on it to a glass slide with antifadent mountant solutions, and observed using a fluorescence microscope(PerkinElmer UltraVIEW Vox).

### Ruffling assay

Ruffling assay was carried out as the method described previously[19]. Briefly, neutrophils were suspended at 1x10^6^/ml in RPMI 1640/10% FBS of indicated pH value, and then stimulated with fMLP at the concentration ranged from 0nM to 10^4^nM. Images were captured every 10sec for 15 min using CCD camera equipped on an inverted microscope(Nikon Ti). The percentage of neutrophils extending pseudopods or ruffling was calculated from fields captured at the indicated time points after chemoattractant stimulation.

### TAXIScan-FL chemotaxis assay

The TAXIScan chamber was assembled with 130μm wide × 4μm thick silicon chip on a 0.17mm cover glass base, as described by the manufacture, and filled with PBS/0.1% BSA. Neutrophils(1μl, 3×10^6^/ml) treated with indicated pH was added to lower reservoir and allowed to line up by sucking the buffer tenderly from the upper reservoir. One microliter of chemoattractant(fMLP, 1μM) was then added to the upper reservoir. Neutrophil migration in each of the channels was captured sequentially every 30sec for 1 hour using 10× lens. The neutrophil migrate tracking, speed and directionality were analyzed using Tracking Tool (Gradientech).

### In vitro phagocytosis assay

FITC conjugated zymosan A(Molecular Probes)were opsonized with 10% human serum at 37°C for 30min. Neutrophils in different pH medium were mixed with these pre-treated bioparticles at a ratio of 1:5(neutrophils: zymosan) and incubated at 37°C for 30min. Negative controls were incubated on ice. Pictures were captured by a fluorescence microscope(600×, Nikon). Phagocytosis efficiency was expressed as the number of the internalized particles per 100 neutrophils(Phagocytosis index). Binding efficiency was expressed as the number of the binding particles per 100 neutrophils(Binding index). More than 100 cells were counted from random fields of each group. *E*.*Coli*(ATCC, strain 19138), were opsonized with 10% human serum at 37°C for 30 min. Purified neutrophils were divided into three groups with 2×10^6^ cells for each one. Each group had three repeats. First, they all co-incubated with the pretreated *E*.*Coli* at a ratio of 1:5 in the pH 7.4 medium for 30 min. Then cells were spined down at 1000 rpm for 5 min. Cell pellet were washed with PBS for 3 times. And then 50ug/ml kanamycin was added to the cells to remove the extracellular bacteria. After 30min at 37°C[20], one group of cells were lysed by adding distilled H_2_O and the diluted aliquots were spread on LB agar overnight at 37°C. The number of CFUs of this group stands for the bacteria loading. At the same time, the other two groups of cell pellets were resuspended using the medium of pH 6.0 and pH 7.4, and incubated at 37°C for another 30 min. After washing with PBS, cells were lysed with distilled H_2_O and the diluted aliquots were spread on LB agar overnight at 37°C. The killing percentage were calculated by the following equation:

$$\text{killing percentage}=\left(1-\frac{\text{CFUs of pH }6\text{.0/7.4}}{\text{bacteria load}}\right)\times 100\%$$

## Results

### 1. Extracellular acidification delay neutrophil apoptosis

**Fig 1:**
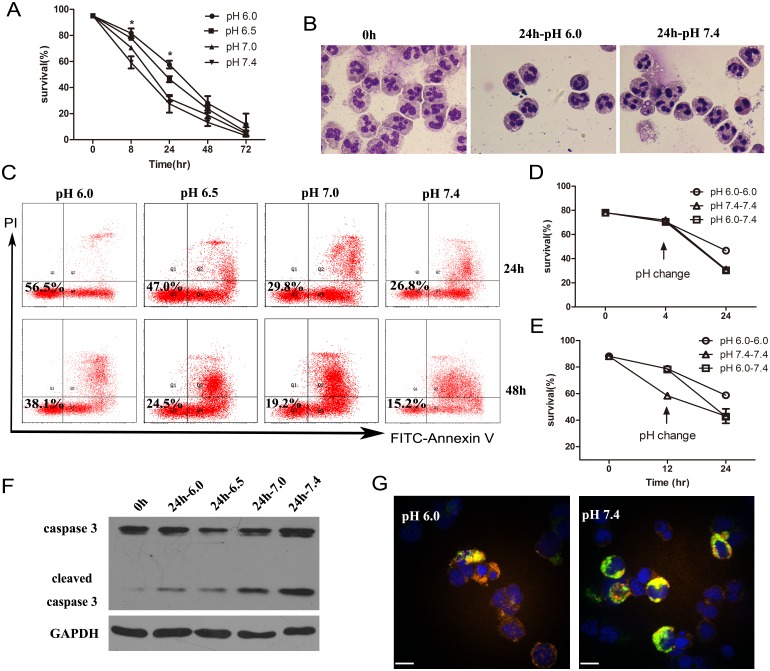
Extracellular acidification delays neutrophil apoptosis and reduces caspase 3 activation. (A) Neutrophils apoptosis is delayed in the acid medium. Freshly prepared neutrophils were cultured in the medium of different pH for indicated periods of time. Cell death was assessed by FACS analysis with annexin V and PI staining. At least three separate experiments were carried out with a minimum of 10,000 cells counted per data point. (B) Light microscopy of a neutrophil population with Wright staining aged for 24h (×1,000). (C) FACS analysis of neutrophil apoptosis. Purified neutrophils were labeled with FITC-annexin V and PI after cultured for indicated time and detected by flow cytometer. (D,E) pH changed from acid to neutral at either the 4^th^ hour(D) or the 12^th^ hour(E) of cell culture will accelerate the neutrophil death. Neutrophils in pH 6.0 were collected and resuspended in the medium of pH 7.4 at the indicated time point for a further culture to 24h. Cell apoptosis was detected by FACS. (F)The cleavage of caspase 3 was decreased when neutrophils were put into the acid medium for 24 hours. Neutrophils were cultured in the medium of pH 6.0 to7.4 for 24h. Protein extracts were resolved on SDS/PAGE. Pro and cleaved caspase 3 were detected by western blot using anti-caspase 3 antibody. (G)The cleaved caspase 3 was observed by immunofluorescence microscope. Immunofluorescence staining was carried out on the neutrophils cutured for 24h. Green stands for the cleaved caspase 3, red for cytoskeleton and blue for nucleus. Scale bar, 7μm. Data are from three independent experiments(A,D,E) or are representative of three experiments(B, C,F,G,). (***,*p*<0.001, **, *p*<0.01).

In this study, we used the flow cytometer analysis combined with Wright Staining to explore neutrophil cell death in different extracellular environment. It is showed that, compared to the normal situation (pH 7.4), neutrophil cell death was delayed when the pH of culture medium was adjusted to acid(pH 7.0, 6.5, 6.0). The percentage of survival cells went up as the pH went down(Fig 1A). After cultured for 24h, no more than 30% of neutrophils were survived (Annexin V^-^/PI^-^) in pH 7.4, but in the pH 6.0 group, the percentage of survival cells increased to more than 55% (Fig 1C). Apoptosis neutrophils showed characteristic morphological changes including nuclear pyknosis, chromatin condensation and cytoplasmic vacuolation[21]. We carried out Wright stain for the neutrophils cultured for 24h and found that compared to the cell in neutral environment, cells in the acid medium showed less nuclear pyknosis and cytoplasmic vacuolation(Fig 1B).

To determine whether or not the delay of neutrophil cell death need sustained extracellular acidification, we changed the medium in the process of neutrophil culture at the 4th or 12th hour. The results suggested than only sustained extracellular acidification can delay neutrophil death. Once back to the normal pH(pH 7.4), the pace of cell death also went back to normal, as the same to pH 7.4(Fig 1D and 1E).

At the same time, we also detected the activation of caspase 3, the executive of cell apoptosis. We can see from the western blot that the cleavage of caspase 3 decreased when the neutrophils were treated in the acid medium for 24 hours(Fig 1F). The immunofluorescence staining for cleaved caspase 3 showed the same result(Fig 1G). It suggested that extracellular acid reduced caspase 3 activation.

### 2. Superoxide production of neutrophils was inhibited in acid environment

**Fig 2:**
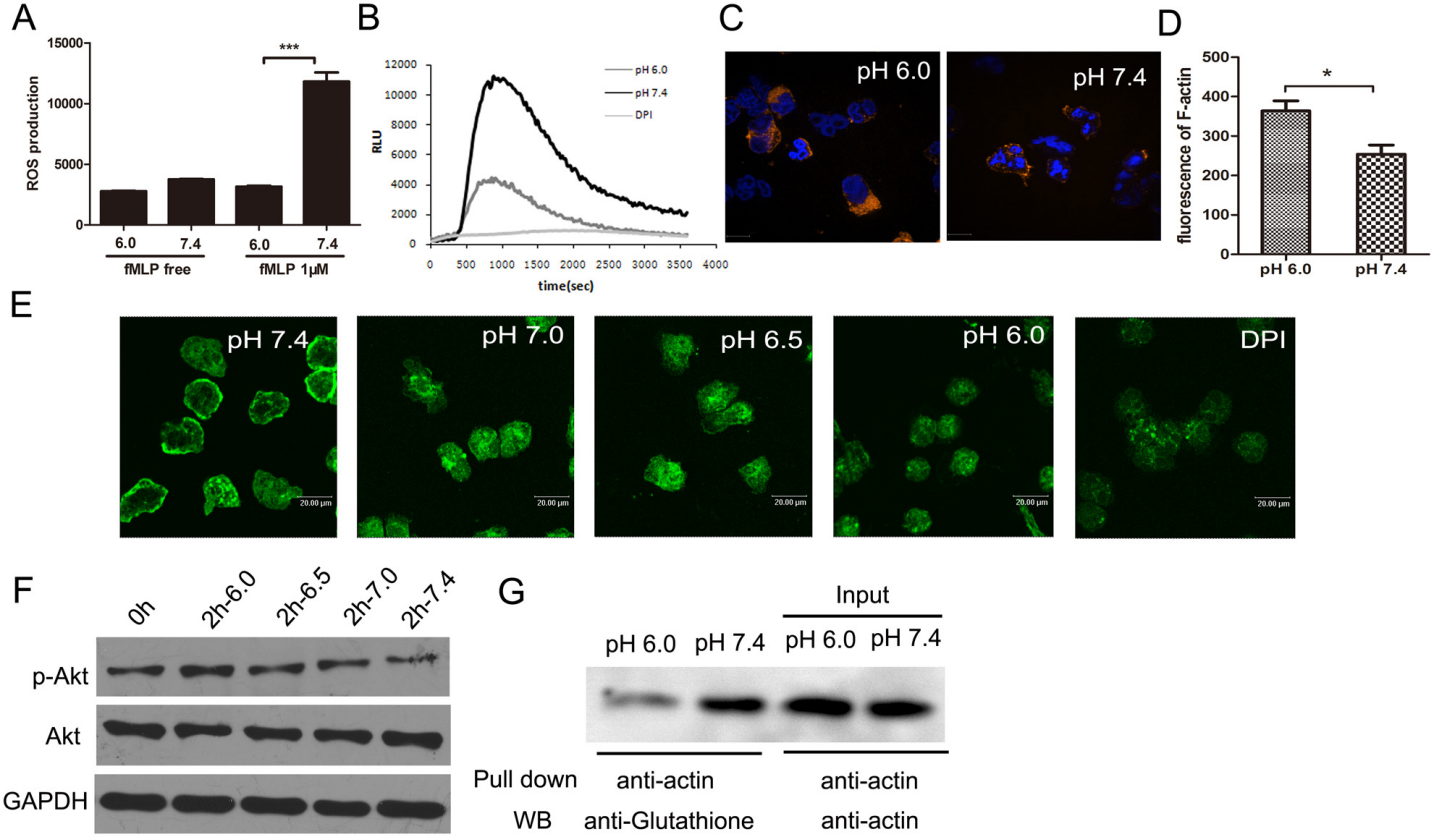
Superoxide production of neutrophils was inhibited in acid environment. (A, E)Fluorescent probe DCFH-DA. 5×10^5^ cells were labeled with DCFH-DA and stimulated by 1μM fMLP for 10 min. Fluorescence intensity were read by the Microplate Reader(A) and images were captured using Leica laser scanning confocal microscope (E). (B)Luminol chemiluminescence assay of neutrophils ROS production. Purified neutrophils in different pH medium were stimulated by 1μM fMLP in the presence of lunimol (50μM) and horseradish peroxidase(HRP, 4U/ml). ROS production was monitored in a luminometer at 37°C. Cells pre-treated with 25μM DPI were as negative control. (C, D) Quantification of F-actin of neutrophils in acid and neutral medium. At the 3^th^ minute of fMLP(10nM) stimulation, cells were fixed and stained with rhodamine-phalloidin(Red) and DAPI(Blue), the fluorescence intensity of rhodamine-phalloidin was read by the Microplate Reader(D). Pictures were taken by fluorescence microscope(C). (F)Extracellular acid increase the Akt phosphorylation of neutrophils Purified neutrophils were cultured in the medium of pH 6.0 to7.4 for 2h, Protein extracts were resolved on SDS/PAGE. Total and phosphorylated Akt were detected by Western blot using anti-Akt and anti-phospho-Akt (Ser473) antibodies. (G) The glutathionylation of actin was down regulated in neutrohils in acid environment. Neutropihls cultured for 4h were collected and lysed by RIPA. Anti-actin antibody and protein A/G agrose beads were used to pull down and anti-glutathione antibody were used in the western blot to detect the glutathionylated actin. Data are from three independent experiments(A,D) or are representative of three experiments(B,C, E,F,G). (***,*p*<0.001, *,*p*<0.05).

It is reported that reactive oxygen species accumulation in neutrophils was responsible for neutrophil death[22, 23]. In order to illuminate if the delay of neutrophil death in extracellular acid was related to ROS, we first detected the ROS production of neutrophils in acid and neutral pH environment by the method of both fluorescent probe and luminol chemiluminescence assay. As it is shown in Fig 2A, under the stimulation of 1μM fMLP, the ROS production raised remarkably in pH 7.4, however, in pH 6.0, it is almost maintained the same level with fMLP free group and raise up very little(Fig 2A). The pictures of laser scanning confocal microscope showed that the ROS production decreased as the pH declined(Fig 2E). We also performed the luminol chemiluminescence assay to detect the ROS production of neutrophils in extracellular acidification. It is showed the same result that respiratory burst was inhibited in neutrophils treated with acid medium(Fig 2B). In order to exclude the influence of pH on the fluorescent and luminescent probe, we carried out a positive control with 10μM H_2_O_2_, and there was very few difference between pH conditions(S2 Fig).

Xu *et al*. have proposed a signal pathway model of neutrophil spontaneous death involved in ROS, actin and PI3K[22]. They claimed that accumulation of ROS might decrease the F-actin level and down regulated PI3K/Akt signal pathway, further caused neutrophil death. On this basis, in order to explore whether the delaying of neutrophils death induced by extracellular acidification has a relationship with this pathway, we tried to figure out the influence of extracellular acid on the actin assemble in neutrophils. First we can see from the immunofluorescence staining that, under stimulation of fMLP, neutrophils in pH 6.0 showed a higher level of F-actin (Fig 2C and 2D). It means that extracellular acid could regulate the F-actin assembly. Studies suggested that F-actin polymerization can be regulated by S-glutathionylation on the cys473 of G-actin, the glutathionylation of G-actin on the thiol groups, often block the activity of assemble to F-actin. Therefore, further immunoprecipitation and western blot assay were performed and the results showed that the glutathionylation of G-actin decreased in acid environment (Fig 2G). Besides, according to the model proposed by Xu, et al. we also detected the Akt phosphorylation. The western blot showed that the phosphorylation of Akt was up regulated in the neutrophils of acid environment. This results was in accordance with a previously study[24]. In conclusion, extracellular acid could inhibit the activity of NADPH oxidase and reduce the ROS production, down regulation the glutathionylation of G-actin and promote F-actin assembly, meanwhile, slow down the inactivation of PI3K/Akt pathway.

### 3. Neutrophils in acid medium were more sensitive to the fMLP induced polarization

**Fig 3:**
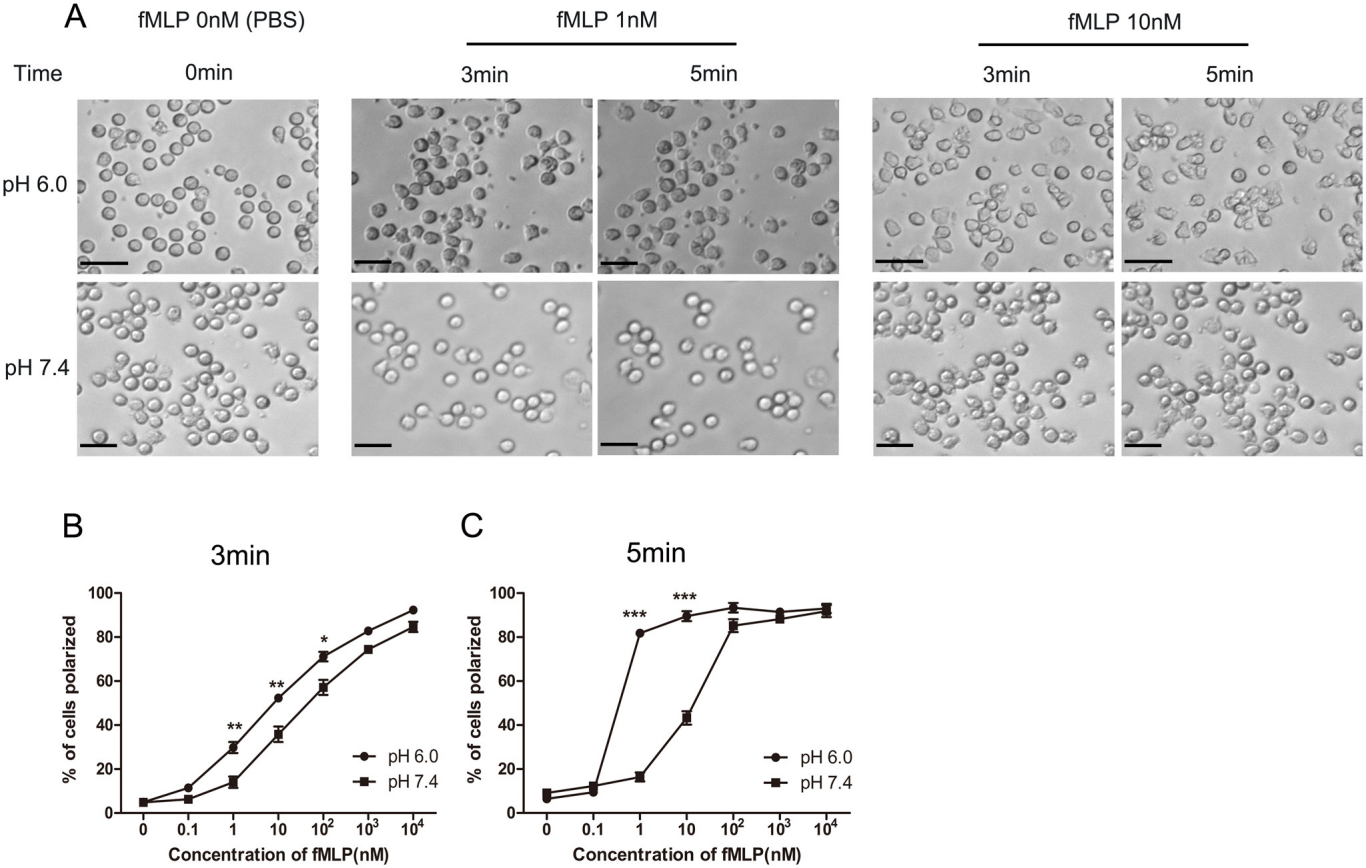
Neutrophils in acid medium were more sensitive to the fMLP-induced polarization. (A) fMLP (concentration ranged from 0nM to 10μM,) induced ruffling in neutrophils cultured in medium of pH 6.0 and pH 7.4. Images were captured every 5 sec for 10 min. (B, C)At 3 min and 5 min of fMLP stimulation, percentage of polarization cells were calculated in each group of different fMLP concentration. Data are from three independent experiments(B, C) or are representative of three experiments(A). (***, *p*<0.001, **, *p*<0.01, *, *p*<0.05).

When neutrophils are exposed to a chemoattractant such as fMLP, they display membrane ruffles and polarize, forming distinct pseudopods and uropods[19]. Thus we next investigated whether extracellular acidification can alter chemoattractant-induced morphologic changes of neutrophils. Unprimed neutrophils were predominately round before stimulation no matter in pH 7.4 or pH 6.0 medium. When a series of concentration of fMLP ranged from 0nM to 10μM were added, neutrophils in different medium began to show different sensitivity(Fig 3A). When the concentration of fMLP was 0.1nM, there was almost no difference between cells in 6.0 and 7.4 in polarization. When the concentration of fMLP was 1nM, neutrophils in pH 7.4 medium showed little morphologic changes (14%) until 5 min after stimulation, while, in the pH 6.0 group, nearly 30% of neutrophils displayed membrane ruffles at the 3^th^ min, and this number raised to about 80% at the 5^th^ min. When the concentration of fMLP increased to 10nM, the pH 7.4 group showed 57% transformation, while the pH 6.0 group had more than 70%. This difference was further increased at the 5^th^ min(Fig 3B and 3C). This suggested that neutrophils in extracellular acid environment were generally more sensitive to chemoattractant than that in neutral medium.

### 4. Extracellular acidification altered neutrophil chemotaxis

**Fig 4:**
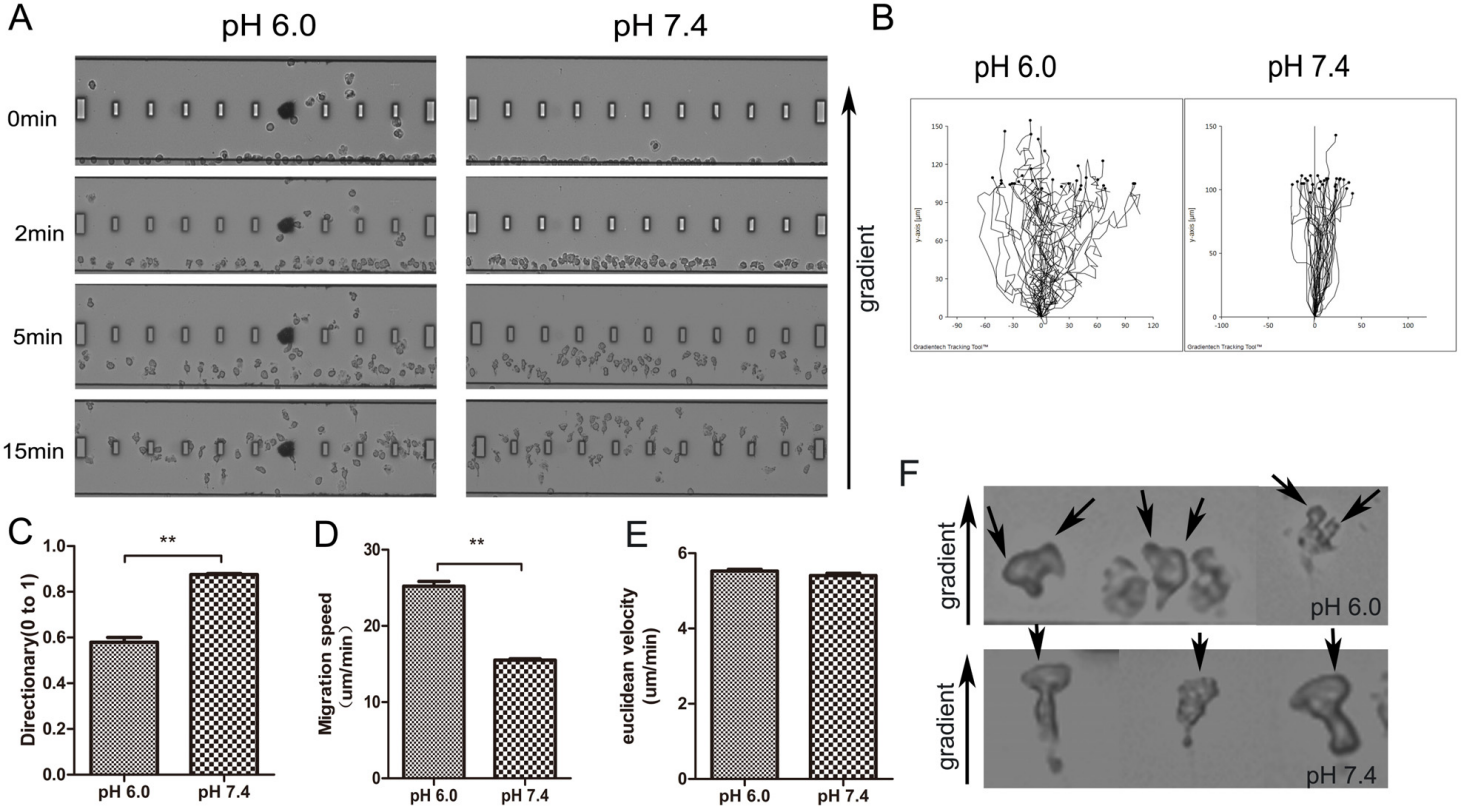
Extracellular acidification altered neutrophil chemotaxis. (A) The screening of neutrophils chemotaxis process. 3000 cells were plated into the TAXIScan-FL device and exposed to shallow chemoattractant gradient generated by addition of 1μl fMLP (1μM). (B) Cell tracks of migrating neutrophils were traced from captured images by the Tracking Tool software (Gradientech). More than 30 cells were picked randomly and analysed. (C, D, E) The directionality and speed were auto-analyzed by the Tracking Tool software (Gradientech). (F) Representative images of migrating neutrophils, black arrowsheads specify pseudopodia. Data are representative of three experiments (**, *p*<0.01).

As neutrophils showed a more sensitivity to fMLP-induced polarization in acid environment, we speculated that it may also have impact on the neutrophil chemotaxis. Therefore, we used the TAXIScan-FL chemotaxis device in which a stable chemoattractant gradient was formed in a 130μm-wide channel. It took about 20min for most of the cells to cross the channel, but there were some difference in the cell tracking between neutrophils in different medium(Fig 4A, S1 Movie). More than 30 cells were picked randomly from each group and the tracking of these cells were drawn by the Gradientech Software(Fig 4B). According to the quantitative analysis of the cell tracking, it is obviously that neutrophils in acid environment displayed defected chemotaxis directionality (Fig 4C). Neutrophils in different environment had almost the same euclidean velocity(Fig 4E), but cells in acid medium went through a longer path in the same time, so these cells presented a faster migration speed(Fig 4D). Focusing on the detail of each cell, we can easily found that neutrophils in acid medium displayed multiple pseudopods(Fig 4F).

### 5. Extracellular acid enhance neutrophil endocytosis while suppress the bacteria killing capability

**Fig 5:**
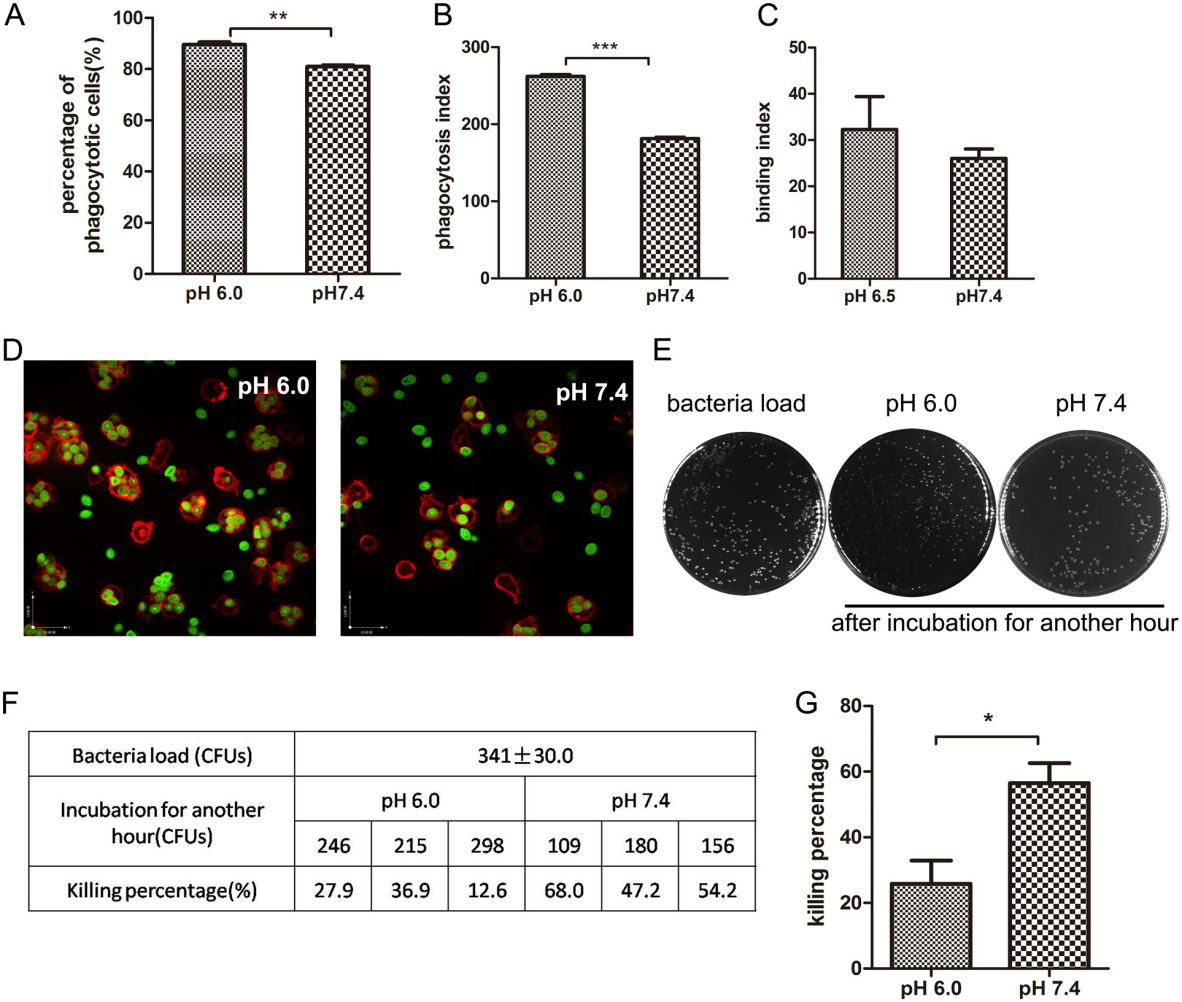
Extracellular acid enhance neutrophil endocytosis but suppress the bacteria killing ability. (A,B,C,D) Neutrophils endocytose FITC-Zymosan. 2×10^6^ neutrophils were mixed with 1×10^7^ opsonized FITC-Zymosan and co-incubated at 37°C for 30 min. Pictures were captured by a fluorescence microscope(600×). (A) Percentage of neutrophils happened to phagocytose. More than 100 cells were counted from random fields of each group. (B) Phagocytosis index was expressed as the number of the internalized particles per 100 neutrophils. (C) Binding index was expressed as the number of the binding particles per 100 neutrophils. (D) Fluorescence images of neutrophil phagocytosis. (E,F,G)Killing assay using neutrophils incubated with *E*.*Coli*.2×10^6^ neutrophils were mixed with 1×10^7^ opsonized *E*.*Coli* and co-incubated at 37°C for 30min. (E) Cells containing *E*.*Coli* were lysed, diluted and spread on the LB agar. CFUs of this group stand for the bacteria loading. Another two groups of neutrophils containing *E*.*Coli* were further incubated in the medium of pH 6.0 and pH 7.4 separately. After incubated for another 30 min, cells were lysed, diluted and spread on the LB agar. (F) The number of CFUs of each group were listed in the table. (G)$\text{killing percentage}=\left(1-\frac{\text{CFUs of pH 6.0/7.4}}{\text{bacteria loading}}\right)\times 100\%$. Data are from three independent experiments(A,B,C) or are representative of three experiments(D,E,F,G,H,I). (***,*p*<0.001, **, *p*<0.01, *,*p*<0.05).

Next we detected the influence of extracellular acid on the phagocytotic ability of neutrophils. Generally speaking, an integrated process of pagocytosis includes two steps: endocytosis and intracellular digestion. In order to detect the endocytosis function, FITC labeled zymosan was used to incubate with neutrophils in acid and neutral medium. 30min later, we can see from the pictures(Fig 5D) that, compared to the cells in neutral medium, neutrophils in acid medium showed an enhanced phagocytosis efficiency, the percentage of phagocytotic cells are much more than the pH 7.4 group(Fig 5A). In addition, through quantifying the average number of bioparticles engulfed by every 100 neutrophils (phagocytosis index), we can see that neutrophils in acid medium had a dramatically increased phagocytosis index: nearly 250 bioparticles were engulfed by 100 neutrophils compared to 180 of pH 7.4 group(*p*<0.0001)(Fig 5B). Meanwhile, the binding index of neutrophils in acid environment was also higher than pH 7.4 group(Fig 5C).

As to the intracellular bacteria killing ability, we carried out the in vitro bacteria killing assay. From the calculated killing percentage, it is clear that under the acid environment, the intracellular killing ability decreased more than 50% compared to the normal condition(pH 7.4)(Fig 5E, 5F and 5G). These results revealed that although neutrophils in acid medium have enhanced endocytosis,the bacterial killing capability is suppressed.

## Discussion

Here we had a systematically screening on the neutrophil functions modulated by extracellular acidification, the results indicated that the low pH value can delay neutrophil spontaneous death, inhibit reactive oxygen species production, enhance neutrophil endocytosis while suppress killing ability, increase their sensitivity to chemoattractant induced polarization and change their chemotaxis.

Extracellular acidification can rapidly cause the decrease of intracellular pH[25]. In our study, we used HCI solution added to the RPMI 1640 medium, which contained bicarbonate, to adjust the pH value of the medium. In this system, pH changes of the environment can rapidly cause the change of intracellular pH through this reversible reaction: HCO_3_^-^+H^+^≒CO_2_ + H_2_O and the transmembrane diffusion of CO_2_[26]. It is reported that when HCI was added, only in bicarbonate containing solution, can the intracellular pH decreased[27].

Under the normal physiological conditions, aged neutrophils trigger spontaneous cell death witha series of morphological changes and biochemistry alternations. A variety of stimuli, such as fMLP, C5a, LPS, G-CSF could prolong the neutrophil life span [28–30]. Here we found that neutrophil apoptosis was delayed in the acid environment. But this effect is quite different from the effect of the extracellular stimuli mentioned above, because we did not see any morphological changes of the neutrophils in the acid medium. So far, the mechanisms about how the low pH regulating the neutrophil apoptosis haven’t been clearly proved. Diego Martinez et al. claimed that extracellular acidosis induced neutrophil activation by a mechanism dependent on activation of phosphatidylinositol 3-Kinase/Akt and ERK Pathways[24]. But whether this activation is associated with cell apoptosis was still unknown. In our study, we found extracellular acid could reduce the ROS production, down regulation the glutathionylation of G-actin and promote F-actin assembly, meanwhile, slow down the inactivation of PI3K/Akt pathway. Additional experiments are required to determine the relationship between these items and neutrophil apoptosis. In some previous studies, ROS was been recognized as mediator of neutrophil death[22, 31], however, when treated the neutrophils from CGD mouse in which the gp91 subunit of NADPH oxidase was deleted[32]in acid and neutral medium, the survival difference was much smaller than wild type(S3 Fig), which suggested ROS may be one of reasons for the apoptosis delaying in acid environment, but not the only reason.

As the intracellular pH goes down, many intracellular enzymes suffer an activity changes. Researches by Deri Morgan *et al*.[33] suggested that the activity of NADPH oxidase of human eosinophils was pH dependence. In return, the activation of NADPH oxidase can produce amount of protons and cause the decrease of intracellular pH[34, 35]. In our study, the ROS production was suppressed by extracellular acidification under the stimulation of fMLP. By contrast, previous reports have presented an evaluation of hydrogen peroxide produce in response to extracellular pH 6.7 compared to pH 7.4[27]. However, our results are consistent with some other researches[36, 37]. These different results may be caused by differences in experiment system, including buffered system, the type and concentration of stimuli, or detected methods. Specific reasons are hard to explain. In our system, we use methods of both fluorescence probe labeling and luminol chemiluminescence assay to confirm our conclusion that ROS production is suppressed under acid conditions.

Our findings also suggested that extracellular acid not only delayed neutrophil cell death, but also modulated functions including polarization, chemotaxis and phagocytosis. Under the stimulation of fMLP, Neutrophils showed a more rapid polarization, and a higher speed of movement. In this regard, unlike our using the fMLP as a chemoattractant, Zygmond and Hargrove put neutrophils in the pH gradient from 5.0 to 8.0. Interestingly, they found the cells were orientated themselves toward pH 5.0. But it doesn’t mean that the protons can act as an chemoattractant. Instead, the truth is acid-induced release of a chemotactic factor [38]. One possible mechanism might account for the fast response to the stimuli of neutrophils in acid medium is the cytoskeleton re-organization. Neutrophils ruffling requires actin cytoskeleton, especially the specifically localized polymerization of actin filaments underneath the plasma membrane at the leading edge to chemokines[39]. It was showed in our studies that extracellular acid caused the reduced actin glutathionylation and increased F-actin assembly, which might be the reason of alteration.

Neutrophils are one of the main phagocytes innate immune response. Interestingly, extracellular acidification had different effects on the two steps of phagocytosis. It enhanced the endocytosis but suppressed the killing ability. A previously study also showed an increased endocytosis of neutrophils in pH 6.5 and attributed it to the PI3K and ERK pathways[24]. However, according to our results, it seems that the internalized bacterial cannot be digested as normal. It is known that NADPH oxidase derived ROS acts as a powerful weapon in the bacteria killing[40],patients of chronic granulomatous disease with NAPDH oxidase defect cannot conduct efficiently kill the pathogen and are apt to get infection[41]. We also found that neutrophils from CGD mouse showed functional changes similar like neutrophils in acid medium, which suggested that ROS played an important role in regulating neutrophil functions including chemotaxis, ruffling and phagocytosis(S4 Fig, S5 Fig and S6 Fig). But more experiments are still needed to find out whether the declined ROS acts as a key regulator in the functional changes caused by extracellular acid.

In conclusion, our research suggested that extracellular acidification delays neutrophil apoptosis and alters their functions. It reminds us that extracellular acidification acts as an essential modulator of neutrophils. However, until now, although many studies have been focused on this topic, most of them were carried out *in vitro*. As a consequence, more in vivo researches are required, especially, to find a property animal model to further illustrate the molecular mechanisms. In the future, on the one hand, some pH sensitive drugs could be designed to make their delivery and release much precise [42]. On the other hand, as research continued, the regulating of extracellular pH may become an important treatment of disease therapy.

## Supporting Information

S1 Fig Gating of the living neutrophils. (TIF)

S2 Fig The pH of the medium has very few influences on the fluorescent and luminescent probe. (A) Fluorescent probe DCFH-DA. (B) Luminol chemiluminescence assay. (TIF)

S3 Fig The apoptosis of neutrophils from CGD and wt mouse in the medium of different pH. (TIF)

S4 Fig Neutrophils from the CGD mouse were more sensitive to the fMLP stimulation. (TIF)

S5 Fig Disruption of NADPH oxidase leads to chemotaxis defects. (TIF)

S6 Fig Depletion of ROS altered neutrophil phagocytosis. (A, B) CFUs of the diluted supernatant. (C) CFUs of the cell lysate. (D) Killing percentage of CGD and WT neutrophils. (TIF)

S1 Movie Neutrophil chemotaxis in acid and neutral medium. (MP4)
