# Correction: Extracellular Acidification Acts as a Key Modulator of Neutrophil Apoptosis and Functions

**DOI:** 10.1371/journal.pone.0139500

**Published:** 2015-09-25

**Authors:** Shannan Cao, Peng Liu, Haiyan Zhu, Haiyan Gong, Jianfeng Yao, Yawei Sun, Guangfeng Geng, Tong Wang, Sizhou Feng, Mingzhe Han, Jiaxi Zhou, Yuanfu Xu

The Funding section is incorrect. The correct funding information is as follows: This study was supported by grants from the National Basic Research Program of China (2012CB966403 and 2015CB964903), the Chinese National Natural Science Foundation (31271484, 31471116, and 31171431), and the Tianjin Natural Science Foundation (12JCZDJC24600).
