# Supplementary figures and images for: Extracellular Acidification Acts as a Key Modulator of Neutrophil Apoptosis and Functions
Source: PLoS One. 2015 Sep 4;10(9):e0137221. doi: 10.1371/journal.pone.0137221 (PMC4560393; doi:10.1371/journal.pone.0137221)

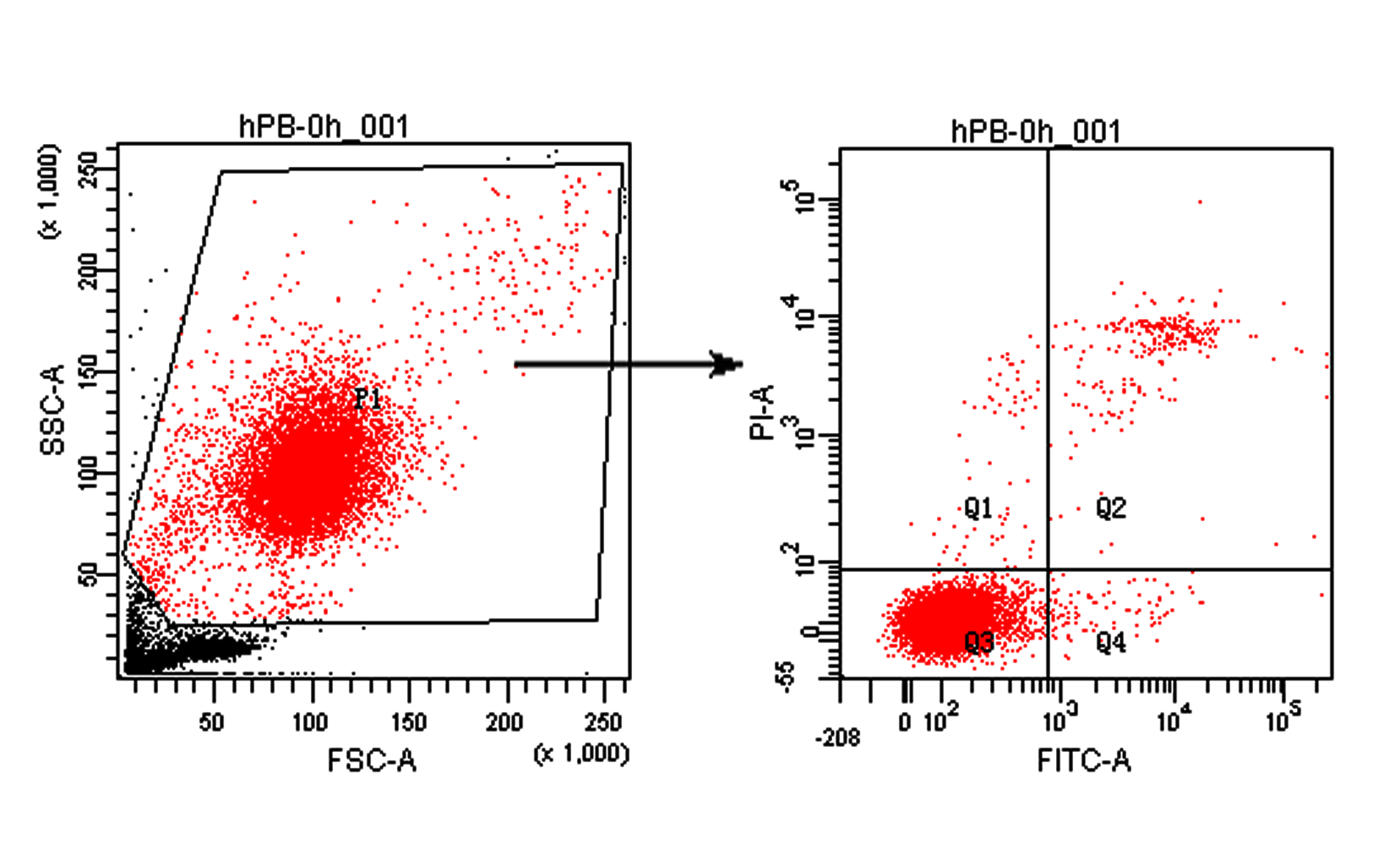

Supplement: S1 Fig — (TIF) [file pone.0137221.s001.tif]

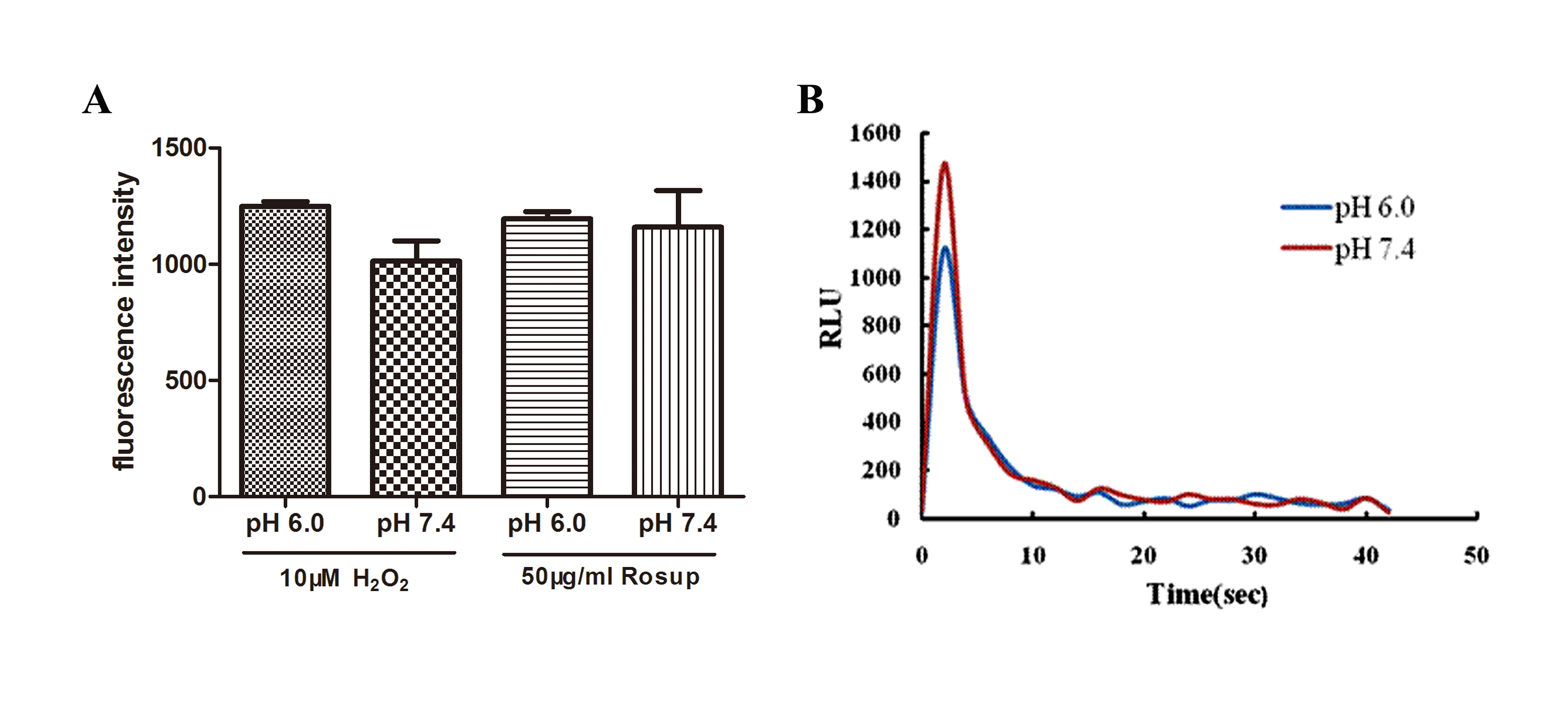

Supplement: S2 Fig — (TIF) [file pone.0137221.s002.tif]

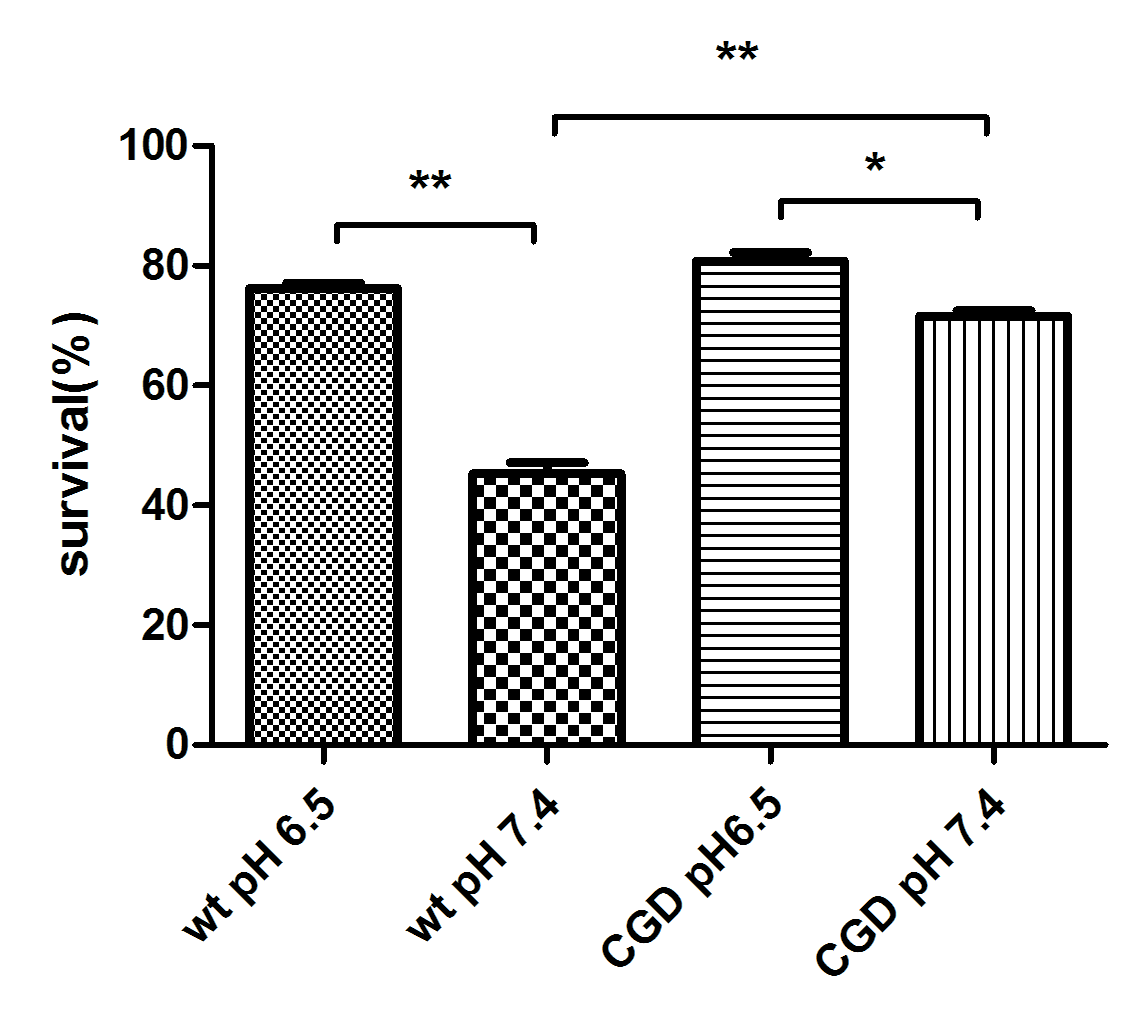

Supplement: S3 Fig — (TIF) [file pone.0137221.s003.tif]

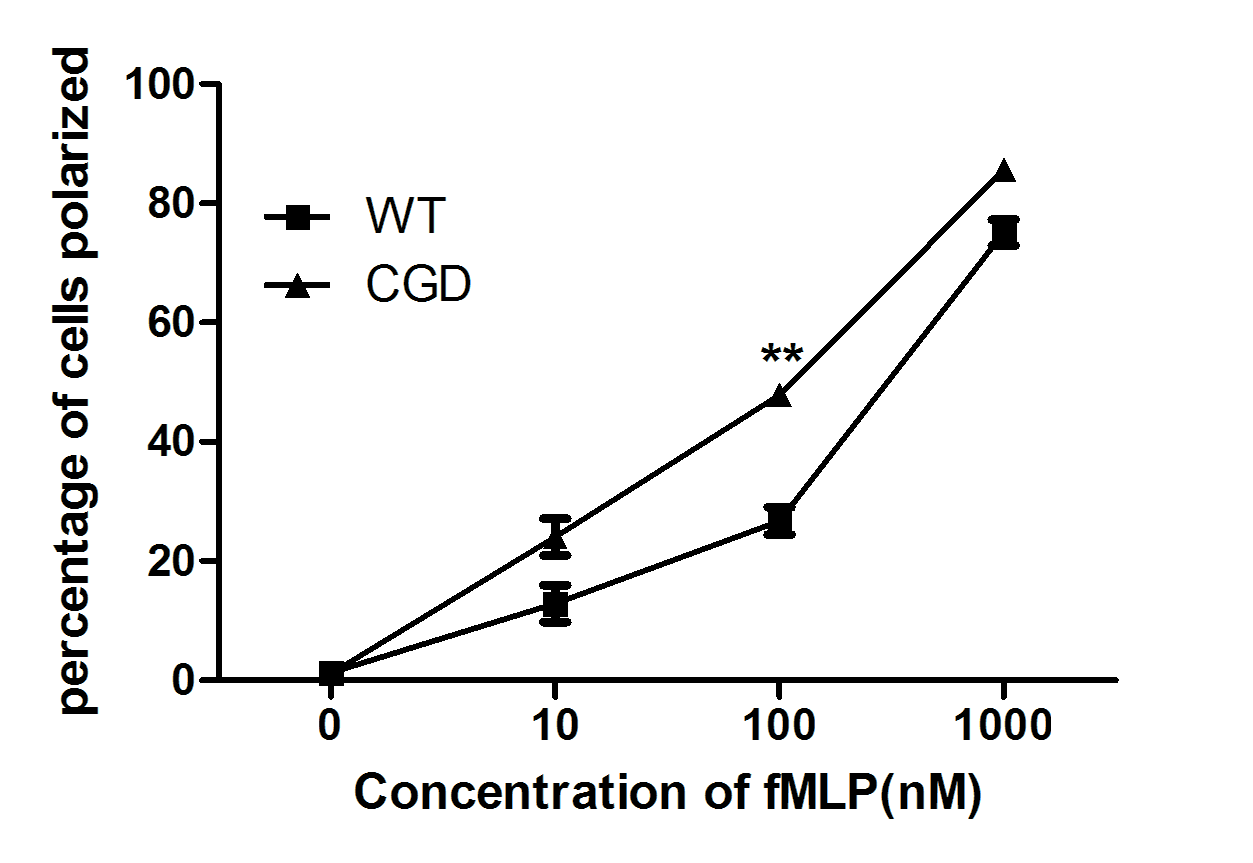

Supplement: S4 Fig — (TIF) [file pone.0137221.s004.tif]

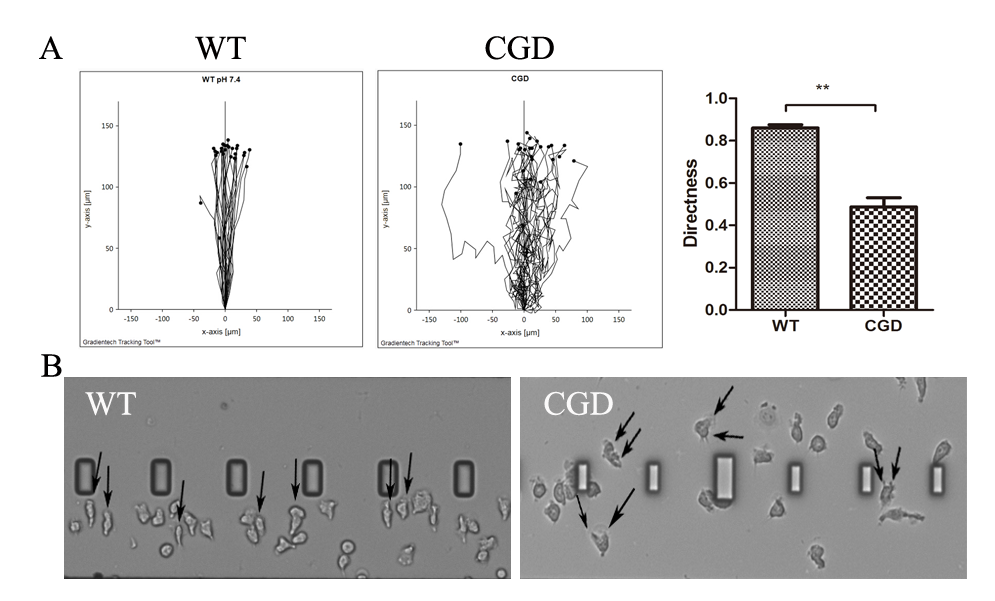

Supplement: S5 Fig — (TIF) [file pone.0137221.s005.tif]

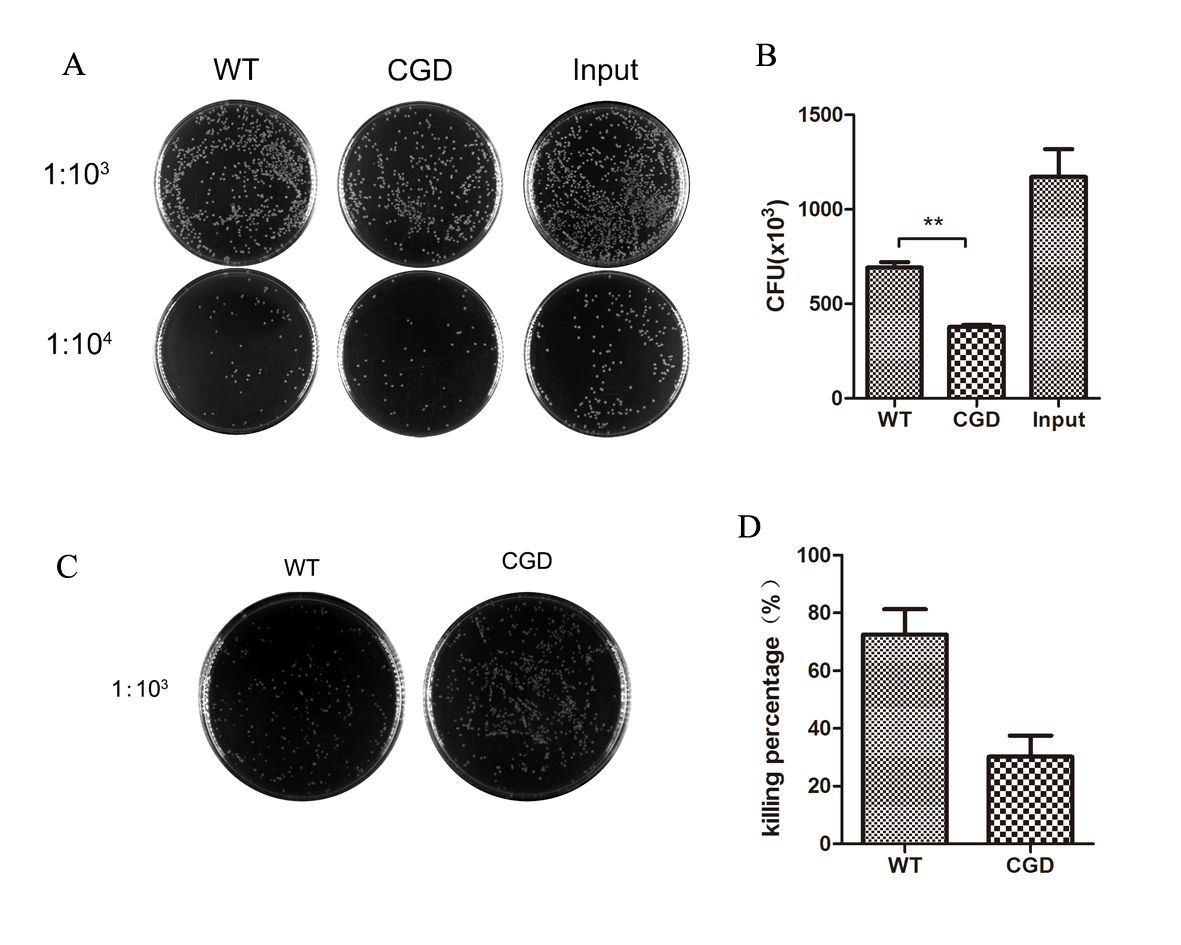

Supplement: S6 Fig — (TIF) [file pone.0137221.s006.tif]
